# Intersectionality of Gender and Social Determinants of Health in Asymptomatic Alzheimer’s Neuropathology

**DOI:** 10.1002/alz.087097

**Published:** 2025-01-09

**Authors:** Lilah M Besser, Deirdre O'Shea, Anthony J. Fuentes, James E Galvin

**Affiliations:** ^1^ University of Miami Miller School of Medicine, Boca Raton, FL USA; ^2^ University of Miami, Miami, FL USA

## Abstract

**Background:**

Studies are limited regarding the role of intersectional social determinants of health in asymptomatic Alzheimer’s disease neuropathology (ADNP).

**Method:**

We analyzed data on 3,107 participants in the National Alzheimer’s Coordinating Center’s Neuropathology Dataset who had a visit ≤24 months before autopsy and confirmed ADNP (moderate to frequent neuritic plaques and Braak Stage III‐VI). Groups were defined based on the presence (symptomatic ADNP, n = 2,956) or absence (asymptomatic ADNP, n = 151) of an MCI/dementia diagnosis at the final visit before death. Social determinants included gender (man/women), educational attainment (years), racialized group (Black, White, other), Hispanic ethnicity, living alone, and English/non‐English primary language. Multivariable logistic regression tested associations between these determinants and ADNP classification (asymptomatic vs symptomatic), and additionally, the models were stratified by gender. Models controlled for age at death, apolipoprotein e4 carrier status, informant‐reported memory decline, time between last visit and autopsy, vascular and Lewy body pathology, and comorbidities (i.e., hypertension, diabetes, depression).

**Results:**

Women (versus men) were more likely to have asymptomatic ADNP (OR = 1.02, 95% CI = 1.00‐1.03), as were those with greater years of education (OR = 1.004, 95% CI = 1.004‐1.006), Hispanic individuals (OR = 1.07, 95% CI = 1.03‐1.11), and those living alone (OR = 1.11, 95% CI = 1.06‐1.17). Non‐English primary language speakers were less likely to have asymptomatic ADNP (OR = 0.88, 95% CI = 0.85‐0.92). Gender‐stratified analyses showed that both women and men were more likely to have asymptomatic ADNP if they were Hispanic (women: OR = 1.07, 95% CI = 1.02‐1.11; men: OR = 1.04, 95% CI: 1.00‐1.08) or living alone (women: OR = 1.12, 95% CI = 1.07‐1.18; men: OR = 1.10, 95% CI: 1.02‐1.18). Women speaking a non‐English primary language were less likely to have asymptomatic ADNP (OR = 0.87, 95% CI: 0.83‐0.91), while in men, more years of education was associated with greater odds of asymptomatic ADNP (OR = 1.004, 95% CI: 1.002‐1.007).

**Conclusion:**

Our study suggests that gender, education, Hispanic ethnicity, primary language, and living alone, as well as the intersectionality of these factors with gender, may differentially influence MCI/dementia diagnosis prior to death in the presence of ADNP. These findings emphasize the need for future Alzheimer’s disease research to prioritize intersectional social determinants of brain health.

